# Effects of Feed Moisture Content on the Physical and Nutritional Quality Attributes of Sunflower Meal-based High-Moisture Meat Analogues

**DOI:** 10.1007/s11947-023-03225-8

**Published:** 2023-10-25

**Authors:** Ravinder Singh, Amanda Gomes Almeida Sá, Shubham Sharma, Mohammad Nadimi, Jitendra Paliwal, James D. House, Filiz Koksel

**Affiliations:** 1 Department of Food and Human Nutritional Sciences, Richardson Centre for Food Technology and Research, University of Manitoba, Winnipeg, MB R3T 2N2 Canada; 2 Department of Biosystems Engineering, University of Manitoba, Winnipeg, MB R3T 5V6 Canada

**Keywords:** Extrusion texturization, Protein digestibility, Meat alternatives, Microstructure, Soy protein, Amino acid requirements

## Abstract

Adding value to food industry by-products, like sunflower meal (SFM), through their utilization as ingredients in new food products can improve sustainability of food systems. This research investigated extrusion cooking to produce high-moisture meat analogues (HMMAs) made from blends of soy protein isolate and expeller-pressed SFM. The effects of feed moisture content [FMC] (60, 65, and 70%, wet basis) and SFM concentration (37.5, 50, and 62.5%, total blend weight basis) on physical and protein nutritional quality attributes of HMMAs were investigated. The processing temperatures (including cooling die), screw speed and feed rate were kept constant at 60-80-115-125-50-25 °C (from feeder to the die end), 200 rpm and 0.5 kg/h (dry basis), respectively. An increase in SFM concentration and FMC significantly (*p* < 0.05) reduced the mechanical energy requirements for extrusion. Cutting strength and texture profile analysis of HMMAs indicated softer texture with increases in SFM and FMC. X-ray microcomputed tomography analysis revealed that the microstructure of the HMMAs at the centre and towards the surface was different and affected by SFM concentration and FMC. The in vitro*–*protein digestibility corrected amino acid score of the HMMAs ranged between 85 and 91% and did not show significant (*p* < 0.05) changes as a function of FMC or SFM concentration. HMMAs produced from 37.5% SFM at 70% FMC showed no deficiency in essential amino acids for all age categories except for infants, suggesting the high potential of SFM and soy protein blends for creating nutritious meat alternative products. Overall, this work provided valuable insights regarding the effects of soy protein replacement by SFM on the textural, microstructural and nutritional quality of HMMA applications, paving the way for value-addition to this underutilized food industry by-product.

## Introduction

Sunflower meal (SFM) is a by-product of the sunflower oil processing industry. It is the third most produced oil seed meal after soybean and rapeseed, with annual worldwide production of ⁓21.5 million metric tonnes (USDA, 2022) and is a rich source of protein (34–54%) (Bárta et al., 2021; Jia et al., 2022a, b) and phenolic compounds (Alexandrino et al., 2021). Due to its high protein content with promising techno-functional properties (Jia et al., 2022a, b), SFM can potentially be used in value-added plant-protein-rich foods. Currently, SFM mostly finds its usage in animal feed and is underutilized in food products.

Effective utilization of food industry by-products, such as SFM, can play a major role in meeting future protein needs. Although generally considered sustainable compared to animal proteins due to their lower carbon footprint and water requirements (Pimentel & Pimentel, 2003), plant proteins are mostly used in their refined forms (i.e., isolates) for producing plant-based meat alternatives. Partial replacement of protein isolates in blend formulations with SFM may help in further achieving the United Nation’s sustainable development goals (United Nations, 2015) by reducing food wastage, improving food security, and reducing the cost of plant-based meat alternatives.

Among extruded meat alternatives, high-moisture meat analogues [HMMAs] (feed moisture contents [FMC] ≥ 40%) have layered and/or fibrous textures that resemble whole-muscle meats (Samard et al., 2019). With the use of high temperatures and shear in a closed barrel under pressure, extrusion processing provides favorable conditions for the structural changes in proteins that are mainly responsible for these textures (Samard et al., 2019). However, under these harsh processing conditions, some heat-sensitive amino acids, such as lysine, can be lost (Samard & Ryu, 2019a). This negative effect on protein nutritional quality can somewhat be compensated with extrusion’s positive impact on protein digestibility through the destruction of anti-nutritional factors such as trypsin and chymotrypsin inhibitors (Alonso et al., 2000). Therefore, the protein nutritional quality of HMMAs needs to be systematically studied for comparison purposes with animal meat products that they aim to replace.

The growth trend for plant-based proteins has amplified the focus on studying their protein quality, which is determined by the protein digestibility, amino acid profile, and their bioavailability for absorption (Boye et al., 2012; Sá et al., 2020). Currently, in North America, the protein digestibility–corrected amino acid score (PDCAAS), calculated using preschool children (aged 2–5 years) amino acid requirements, is the required method for evaluating the eligibility of foods for protein content claim purposes (FAO/WHO, 1991). However, in 2013, the Protein Evaluation in Human Nutrition Committee proposed a new method, called the digestible indispensable amino acid score (DIAAS), and recommended amino acid scoring patterns for different age categories (FAO/WHO, 2013). Hence, it is important to evaluate the protein quality of plant-based meat alternatives according to these different guidelines for future dietary protein recommendations.

Although in vivo assays are still required for protein content claims, in vitro methods can provide an economical and rapid alternative, and have been shown to strongly correlate with in vivo methods (Nosworthy et al., 2017, 2018a, b). Amino acid profile and in vitro protein digestibility of meat alternatives produced from soy, wheat, and pea proteins have been reported (Lin et al., 2022; Osen et al., 2015; Samard & Ryu, 2019a, b). Baune et al. (2021) studied the amino acid composition of HMMAs produced from commercial sunflower seed protein and PDCAAS of pork patties extended with these HMMAs at a 30% level. However, protein quality evaluation of HMMAs produced from SFM following different PDCAAS and DIAAS guidelines has not been previously carried out.

Regarding the amino acid composition of protein-rich foods, meat products are considered better sources of essential amino acids compared to plant proteins which are generally limited in one or more essential amino acids and therefore deemed incomplete protein sources (Edge & Garrett, 2020). The blending of proteins from different plants has proven to be an effective strategy to improve the nutritional (Li et al., 2023; Nosworthy et al., 2017) and physical (Chiang et al., 2019; Wittek et al., 2021) properties of extruded foods. Soy protein isolate (SPI) is one of the most widely used raw materials for the manufacture of HMMAs due to its easy availability, excellent techno-functional properties, and relatively better essential amino acid composition compared to other plant protein sources (Kumar et al., 2017). Because of its high lysine content (Gorissen et al., 2018), blending soy proteins with SFM which is limited in lysine (Sosulski & Fleming, 1977) can help provide a well-balanced amino acid composition in the end product.

The present study was carried out to investigate the potential of SFM in HMMA applications. This study is the first to include SFM in soy-based extrusion feed formulations for the production of HMMAs. The objectives were to study the effects of SFM concentration (37.5, 50, and 62.5% on total blend weight basis) in blend formulations and extrusion FMC (60, 65, and 70%, wet basis) on the physical properties and protein quality attributes of HMMAs made from blends of SFM and SPI. The physical properties studied were textural quality attributes including cutting strength, degree of texturization, texture profile analysis, and color properties, while the nutritional quality attributes were an amino acid profile, in vitro protein digestibility, in vitro-protein digestibility corrected amino acid score, and in vitro–digestible indispensable amino acid score.

## Material and Methods

### Materials

SPI was purchased from Solbar Ningbo Protein Technology Co., Ltd. (Ningbo, China). SPI was tested for protein content and moisture content using AACC International (1999) methods 46–13 and 44–19.01, respectively. Crude fat and ash contents were analyzed following Min and Ellefson (2010) and Marshall (2010), respectively.

Sunflower seeds were obtained from Turtle Mountain Seed Co. (Deloraine, MB, Canada) and expeller-pressed at the Richardson Center for Food Technology and Research (University of Manitoba, Winnipeg, MB, Canada). Defatted SFM was then pulverized to particle size < 0.75 mm using an impact mill (M-21, Prater Industries, Bolingbrook, USA). The proximate composition analysis of SFM was carried out at the Central Testing Laboratory (Winnipeg, MB, Canada) using AOAC International (2012) methods for moisture content (930.15), protein content (990.03), and ash content (923.03). The crude fat content was measured according to the AOCS method Am 5–04 (2017).

### Extrusion Processing

Three SFM and SPI blend formulations were prepared to achieve 37.5, 50, and 62.5% of SFM in the formula. HMMAs were produced from these blends at three different FMC: 60, 65, and 70 g water/100 g feed (wet basis), using a lab-scale, co-rotating twin-screw extruder (MPF19, APV Baker Ltd., Peterborough, UK). The four temperature-controlled zones of the extruder barrel were set at constant temperatures of 60, 80, 115, and 125 °C from the feeder towards the die end. A constant screw speed of 200 rpm and feed rate of 0.5 kg/h (d.b.) was used. The screw profile was set following Koksel and Masatcioglu (2018). A long cooling die (inside dimensions: 300 × 50 × 5 mm) was attached to the end of the extruder barrel to facilitate the fibrous structure formation in the HMMAs. The long cooling die had two temperature-controlled zones which were set at 50 °C (close to the barrel) and 25 °C (far from the barrel). During extrusion, the torque and die pressure values were recorded in quadruplicates, and specific mechanical energy (SME) input was calculated following Luo and Koksel (2020). The HMMAs were stored in zipped plastic bags at − 18 °C. Approximately, 60–75 g (dry basis) of the HMMAs collected at each condition were freeze-dried (Genesis XL-70, Virtis, Warminster, PA, USA) and milled using a centrifugal mill (ZM200, Retsch, Haan, Germany) to particle size < 0.75 mm for protein quality analyses.

### Texture

#### Cutting Strength and Degree of Texturization

The longitudinal (parallel to the melt flow direction inside the die) and transverse (perpendicular to the melt flow direction inside the die) cutting force of the HMMAs were measured following Osen et al. (2014) with minor modifications. In brief, the HMMAs were first thawed to room temperature and cut into 2 cm × 2 cm pieces (thickness: 5 mm). Each HMMA piece was then cut to 75% of its original thickness using a cutting probe (A/ECB) mounted on a texture analyzer (TA-XT-plus, Stable Micro Systems, Godalming, UK) with a 5-kg load cell. The peak force (*N*) from the force vs. time graph was defined as the cutting force in either the longitudinal or the transverse direction. In each direction, the cutting test was performed in six replications. The degree of texturization was calculated by taking the ratio of the transverse cutting force to the longitudinal cutting force.

#### Texture Profile Analysis (TPA)

The texture profile attributes of the HMMAs were analyzed following Ramos-Diaz et al. (2022) with minor modifications. Using a cylindrical probe of a 38-mm diameter equipped with a texture analyzer (TA-XT-plus, Stable Micro Systems, Godalming, UK) with a 30-kg load cell, two compressions were performed on 2 cm × 2 cm cut pieces of HMMAs (thickness: 5 mm). Textural quality attributes including hardness, springiness, gumminess, and chewiness were obtained from the force vs. time graph. Hardness was defined as the peak force (*N*) in the first compression cycle, and springiness, gumminess, and chewiness were calculated using the following equations (Ramos-Diaz et al., 2022):

1 $$\mathrm{Springiness}=\frac{\mathrm{Time\,to\,reach\,the\,peak\,force\,during\,the\,second\,compression}}{\mathrm{Time\,to\,reach\,the\,peak\,force\,during\,the\,first\,compression}}$$

2 $$\mathrm{Gumminess}= \frac{\mathrm{Area\,of\,second\,compression}}{\mathrm{Area\,of\,first\,compression}} \times \mathrm{Hardness}$$

3 $$\mathrm{Chewiness}=\mathrm{Gumminess}\times \mathrm{Springiness }$$

where area refers to the area under the curve in the force vs. time graph.

### X-ray Microtomography

An X-ray microcomputed tomograph (Skyscan 1275, Bruker, Belgium) was used to investigate the microstructure of the freeze-dried HMMAs. The optimal scanning parameters were determined in preliminary experiments and were as follows: a source voltage of 40 kV, a current of 250 μA, and a rotational angle of 0.2° over 180°. To avoid any vibrations during the rotation of the sample, the specimens were securely positioned on a brass sample holder and affixed with a thin layer of low-density wax (Nadimi et al., 2022). Scan time per extrudate piece was ~ 15 min.

Using the NRecon software (version 1.6.10.2, Bruker, Belgium), the raw X-ray images were reconstructed into cross-sectional images with a resolution in the range of 13–20 μm/pixel. CTAn software (Bruker, Belgium) was utilized to select three cross-sectional images (each 9 mm in diameter) from each freeze-dried extrudate piece: one representing a slice close to the top surface (representing the microstructure within the 5–15% of the sample thickness from the top surface), one close to the center (representing the microstructure within the 45–55% of the sample thickness from the surface), and one close to the bottom surface (representing the microstructure within the 5–15% of the sample thickness from the bottom surface) of scanned HMMA samples.

### Color Analysis

A color spectrophotometer (CM-3500d, Minolta, Osaka, Japan) was used to analyze the color attributes of the HMMAs. The HMMAs were thawed to room temperature, cut into 2 cm × 2 cm (thickness: 5 mm), and placed above the light shuffle of the color spectrophotometer. Lightness (L*), greenness-redness (a*), and blueness-yellowness (b*) were measured in triplicates. Taking the color attributes of raw blend formulations as a reference, the total color change (Δ*E*) was calculated using the following equation:

4 $$\Delta E= \sqrt{{{(L}_{m}- {L}_{r})}^{2}+ {{(a}_{m}- {a}_{r})}^{2}+ {{(b}_{m}- {b}_{r})}^{2} }$$

where *L*_*m*_, *a*_*m*_, and *b*_*m*_ were the color attributes of HMMAs and *L*_*r*_, *a*_*r*_, and *b*_*r*_ were the color attributes of raw blend formulations.

### Protein Quality Assessment

#### Protein Content and Amino Acid Composition

The protein contents of HMMAs were determined using the Dumas combustion method, according to AOAC method 990.03 (AOAC International, 2012). Nitrogen analysis was conducted by Central Testing Labs (Winnipeg, MB, Canada), and 6.25 was used as the conversion factor (*N* × 6.25).

The concentrations of most amino acids except methionine, cysteine, and tryptophan were determined using the acid hydrolysis procedure according to AOAC method 982.30. The performic acid oxidized hydrolysis procedure (method 985.28) (AOAC International, 2012) was used to determine methionine and cysteine. Sample analysis was conducted using a Shimadzu Nexera ultra-high-performance liquid chromatography (UHPLC) system (Kyoto, Japan) equipped with a Waters AccQ C18 column (100 mm × 2.1 mm, 1.7 µm). The column oven temperature was set as 51 °C for regular amino acids and 40 °C and 60 °C for cysteine and methionine, respectively. The regular derivatives were determined by UV detection at 260 nm and the run time was 17 min. For sulfur amino acids, the detection was done by fluorescence with excitation at 266 nm and emission at 473 nm, and the run time was 30 min for each sulfur amino acid. The Lab Solutions software (Shimadzu, Kyoto, Japan) was used to process data from the UHPLC. Tryptophan was determined using alkaline hydrolysis, following the ISO 13904:2005 method (ISO, 2005). The sample was injected into a Phenomenex Luna C18 column (250 mm × 4.6 mm, 3 µm) with a flow rate of 1 mL/min. The running time by reversed-phase UPLC was 34 min. Specific fluorescence detection was applied using an excitation wavelength of 280 nm and an emission wavelength of 356 nm.

#### Amino Acid Score (AAS)

HMMAs’ amino acid composition was used to estimate the AAS as [mg of amino acid in test protein/mg of amino acid in requirement pattern] × 100 (FAO/WHO, 1991). Scoring patterns with different age categories were used: (a) preschool children (2 to 5 years) (FAO/WHO, 1991); (b) infants (birth to 6 months), (c) young children (6 months to 3 years), and (d) older children, adolescents, and adults (FAO/WHO, 2013). For each HMMA, the essential amino acid with the lowest AAS was reported as the first-limiting amino acid and was used to establish the overall score following FAO/WHO (1991).

#### In Vitro Protein Digestibility (IVPD)

The IVPD of the HMMAs was determined by Hsu et al. (1977) pH drop method, with minor modifications (Tinus et al., 2012). While stirring at 37 °C, the pH of the protein suspension (6.25 mg/mL) was adjusted to 8.0 with 0.1 N NaOH or 0.1 M HCl. One milliliter of multienzyme solution contained 1.6 mg of trypsin (porcine pancreatic trypsin type IX-S, 13,000–20,000 Na-benzoyl-L-arginine ethyl ester (BAEE) units/mg protein, T0303, Sigma-Aldrich, St. Louis, MO, USA), 3.1 mg of α-chymotrypsin (bovine pancreatic chymotrypsin type II, ≥ 40 units/mg protein, C4129, Sigma-Aldrich, St. Louis, MO, USA), and 1.3 mg of peptidase (protease from streptomyces griseus type XIV, P3.5 units/mg solids, P5147, Sigma-Aldrich, St. Louis, MO, USA). The multienzyme solution was maintained in an ice bath and adjusted to pH 8.0. The enzymatic solution was added to the protein solution at a 1:10 v/v ratio and stirred at 37 °C. After 10 min, the pH mixture was measured using a pH meter. IVPD as a percentage of digestible protein was estimated according to pH variation after 10 min (${\Delta \mathrm{pH}}_{10 \mathrm{min}}$), as shown in the following equation:

5 $$\mathrm{IVPD}\,\left(\%\right)=65.66+18.10\times {\Delta \mathrm{pH}}_{10 \mathrm{min} }$$

#### In Vitro–Protein Digestibility Corrected Amino Acid Score (IV–PDCAAS) and In Vitro–Digestible Indispensable Amino Acid Score (IV–DIAAS)

The IV–PDCAAS was calculated as the product of the lowest AAS and IVPD values for each sample evaluated, using the preschool children (2 to 5 years) scoring pattern (FAO/WHO, 1991). The IV–DIAAS was estimated using different FAO/WHO (2013) scoring patterns: infants (birth to 6 months), young children (6 months to 3 years), and older children, adolescents, and adults. Also, the FAO/WHO (2013) report recommended that in the absence of ileal digestible amino acid values, true fecal protein digestibility can be used. Thus, given the absence of individual digestible amino acid values in this work, IVPD values were used to estimate IV–DIAAS.

### Statistical Analysis

One-way ANOVA using Tukey’s standardized test (*p* < 0.05) was performed on SAS software (Version 9.4, SAS Institute Inc., Cary, NC, USA) to determine the significant differences between different treatments.

## Results and Discussion

### Proximate Composition

SPI contained 87.55% protein content (d.b.), 2.64% fat content (d.b.), and 4.68% ash content (d.b.), while SFM contained 47.94% protein content (d.b.), 12.04% fat content (d.b.), and 7.95% ash content (d.b.). The protein content of the blend formulations decreased with the increased concentration of SFM as the calculated protein content (d.b.) of blend formulations containing 37.5% SFM, 50% SFM, and 62.5% SFM was 72.69%, 67.73%, and 62.78%, respectively. In contrast, the fat content of the blend formulations increased as a function of SFM concentration as the calculated fat content (d.b.) of blend formulations containing 37.5% SFM, 50% SFM, and 62.5% SFM was 6.17%, 7.34%, and 8.52%, respectively.

### Torque, Pressure, and Specific Mechanical Energy (SME) During Extrusion

The torque, die pressure (i.e., the pressure measured at the entrance of the long cooling die), and SME values during extrusion as a function of SFM concentration and FMCs are presented in Table 1. Torque, die pressure, and SME significantly (*p* < 0.05) decreased with an increase in SFM concentration. The only exception to this trend was for extrusion processing at FMC of 70% when SFM concentration was increased from 50 to 62.5% where the decrease in torque, die pressure, and SME values were not statistically significant (*p* < 0.05). The lower torque, die pressure, and SME at higher SFM concentration may be attributed to the higher oil and lower protein content of the blend formulations containing higher SFM. The oil acts as a lubricant and reduces the viscosity of the melt, consequently decreasing the shearing forces and die pressure during the extrusion processing (Kendler et al., 2021; Wang et al., 2022). Similarly, due to decreased chance of protein cross-linking at relatively lower protein concentrations (Kantanen et al., 2022), lower protein levels require less mechanical energy (i.e., SME) input during extrusion cooking (Palanisamy et al., 2019). Similar trends in torque and die pressure as a function of the protein content of blend formulations were reported during high-moisture extrusion of faba bean protein concentrate and isolate mixtures (Kantanen et al., 2022).

**Table 1:** Effects of sunflower meal (SFM) concentration and feed moisture content (FMC) on torque, die pressure, and specific mechanical energy (SME) input during extrusion processing

**SFM concentration (%)**	**FMC** **(%)**	**Torque** **(%)**	**Die pressure (kPa)**	**SME** **(Wh/kg)**
	**60**	12.0 ± 0.0^a^	1712.5 ± 83.5^a^	84.5 ± 0.0^a^
**37.5**	**65**	10.8 ± 0.5^b^	1225.0 ± 88.6^b^	66.2 ± 2.9^d^
	**70**	9.0 ± 0.0^d^	625.0 ± 46.3^e^	47.5 ± 0.0^ g^
	**60**	11.0 ± 0.0^b^	1275.0 ± 88.6^b^	77.4 ± 0.0^b^
**50**	**65**	9.9 ± 0.4^c^	775.0 ± 46.3^d^	60.8 ± 2.2^e^
	**70**	8.0 ± 0.0^ef^	412.5 ± 64.1^f^	42.2 ± 0.0^ h^
	**60**	10.0 ± 0.0^c^	1050.0 ± 53.5^c^	70.4 ± 0.0^c^
**62.5**	**65**	8.4 ± 0.5^e^	525.0 ± 46.3^e^	51.6 ± 3.2^f^
	**70**	7.9 ± 0.4^f^	362.5 ± 74.4^f^	41.6 ± 1.9^ h^

Data is expressed as mean ± standard deviation, *n* = 2 for extrusion runs and *n* = 4 for torque, die pressure, and SME values. Different letters in a column are significantly different (*p* < 0.05)

In terms of FMC, the torque, die pressure, and SME values significantly (*p* < 0.05) decreased with an increase in FMC. Similar to oil, water also acts as a lubricant during extrusion processing and higher FMC generally reduces the viscosity of the melt inside the extruder barrel (Chen et al., 2010), thereby reducing the extrusion system parameters (Palanisamy et al., 2019; Saldanha do Carmo et al., 2021; Singh & Koksel, 2021). An increase in die pressure may reflect the back pressure created in the die by the increased melt viscosity (Osen et al., 2014). Likewise, Chen et al. (2010) reported an increase in die pressure with a decrease in FMC.

### Texture

**Fig. 1:**
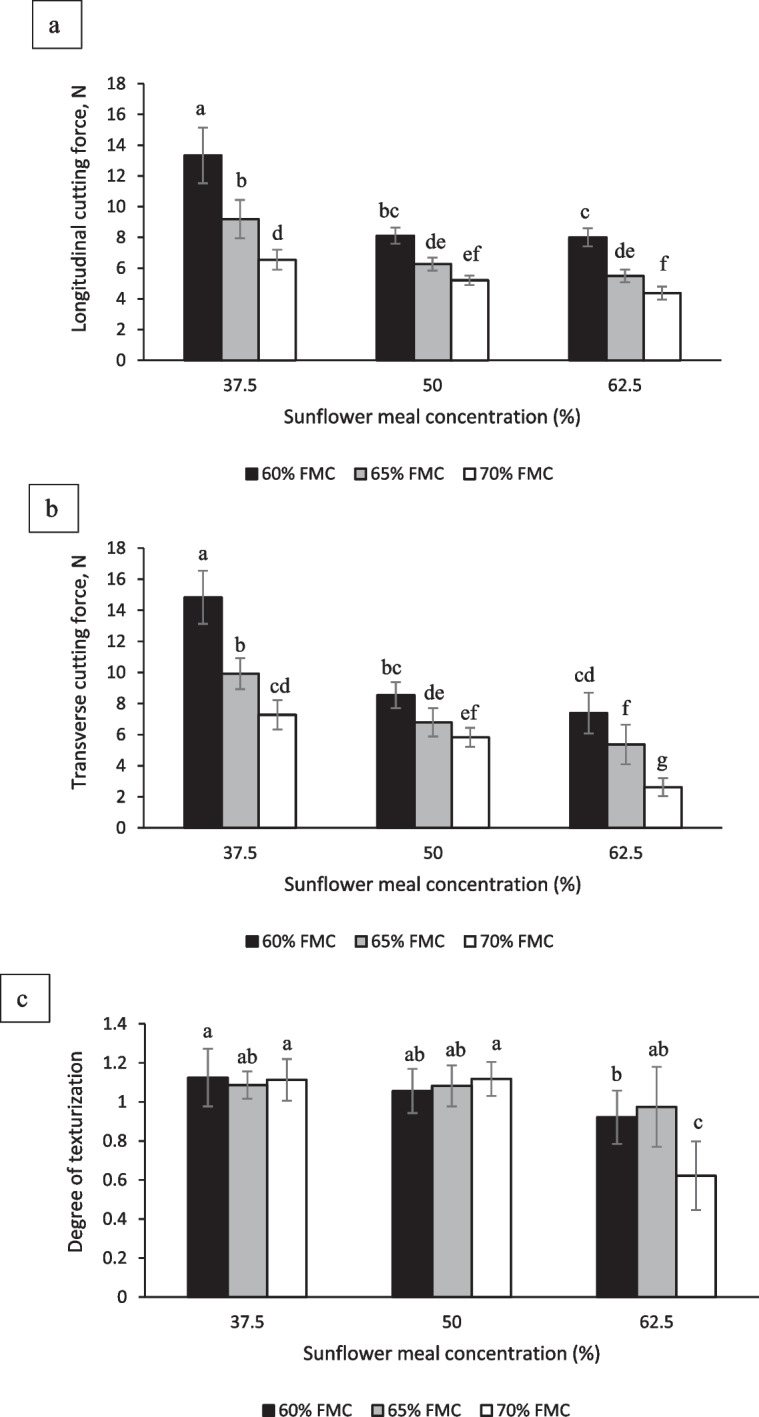
Effects of sunflower meal (SFM) concentration and feed moisture content (FMC) on **a** longitudinal cutting force, **b** transverse cutting force, and **c** degree of texturization of high-moisture meat analogues. Errors bars depict ± standard deviation (*n* = 2 for extrusion runs, *n* = 6 for longitudinal cutting, transverse cutting, and degree of texturization). Different letters on bars in each sub-figure reflect significant differences (*p* < 0.05)

The results for the longitudinal and transverse cutting force of HMMAs are presented in Fig. 1a, b, respectively, and TPA attributes are presented in Table 2. Regardless of the SFM concentration, an increase in FMC resulted in a significant (*p* < 0.05) decrease in cutting force (both longitudinal and transverse) and TPA attributes, which is in agreement with the literature on HMMAs made from hemp protein (Rajendra et al., 2023), soy and hemp protein mixtures (Zahari et al., 2020), faba bean protein (Kantanen et al., 2022), and lupin protein (Palanisamy et al., 2019). The higher water content of the HMMAs produced at higher FMC is the most probable reason for their softer texture (Lin et al., 2000). Higher FMC reduces the relative concentration of protein in the protein melt inside the extruder barrel and the long cooling die, hence providing fewer protein molecules for cross-linking. Moreover, higher FMC may also result in inefficient texturization of the protein melt due to less shear and friction inside the long cooling die which can also reduce HMMA hardness (Lin et al., 2000).

**Table 2:** Effects of sunflower meal (SFM) concentration and feed moisture content (FMC) on the texture profile analysis attributes, i.e., hardness, springiness, gumminess, cohesiveness and chewiness of high-moisture meat analogues

**SFM concentration** **(%)**	**FMC** **(%)**	**Hardness** **(N)**	**Springiness**	**Gumminess** **(N)**	**Chewiness** **(N)**
**37.5**	**60**	170.2 ± 12.7^a^	1.0 ± 0.1^a^	134.3 ± 12.1^a^	133.9 ± 10.8^a^
	**65**	151.3 ± 17.4^b^	0.9 ± 0.1^bc^	121.6 ± 14.6^b^	111.2 ± 17.8^b^
	**70**	114.3 ± 10.2^d^	0.9 ± 0.0^ cd^	87.8 ± 6.0^d^	76.8 ± 5.6^d^
**50**	**60**	134.3 ± 8.2^c^	1.0 ± 0.0^ab^	102.4 ± 8.2^c^	97.9 ± 8.9^c^
	**65**	120.7 ± 6.1^d^	0.9 ± 0.1^ cd^	90.4 ± 5.7^d^	78.3 ± 7.7^d^
	**70**	90.8 ± 6.0^e^	0.8 ± 0.0^d^	69.3 ± 4.9^e^	58.2 ± 4.5^e^
**62.5**	**60**	137.5 ± 8.9^c^	0.9 ± 0.1^bc^	104.9 ± 8.8^c^	95.8 ± 7.2^c^
	**65**	109.6 ± 4.8^d^	0.9 ± 0.0^ cd^	83.5 ± 3.6^d^	72.9 ± 4.8 ^d^
	**70**	51.6 ± 5.3^f^	0.9 ± 0.0^bc^	36.9 ± 3.8^f^	33.5 ± 3.6^f^

Data is expressed as mean ± standard (*n* = 2 for extrusion runs and *n* = 6 for texture tests). Values followed by different letters in a column are significantly different (*p* < 0.05)

In terms of the SFM concentration of blend formulations, regardless of the FMC, an increase from 37.5 to 62.5% SFM significantly (*p* < 0.05) decreased the cutting force in both longitudinal and transverse directions (Fig. 1a, b, respectively), as well as the hardness, chewiness, and gumminess (Table 2). The softer and less chewy texture of HMMAs produced at higher SFM concentration may make them a better option for elderly population as they generally have difficulties consuming harder and chewy products (Liu et al., 2022). The fibrous structure of the HMMAs is mainly formed by protein–protein interactions (Chen et al., 2011; Liu & Hsieh, 2008). The higher SFM concentration decreased the relative protein content in the blend formulations which probably reduced the extent of protein cross-linking during extrusion and thus may be responsible for the relatively softer structures observed. Similar trends in texture attributes with a decrease in protein content of blend formulations have previously been reported for meat alternatives produced using high-moisture extrusion processing of faba bean protein (Kantanen et al., 2022) and low moisture extrusion processing of pea and oat protein blends (De Angelis et al., 2020). Another possible reason for differences in textural attributes as a function of SFM concentration may be the relatively higher oil content of SFM compared to SPI. The strength of protein networks may reduce at higher oil content due to a decrease in protein polymerization reactions (Kendler et al., 2021). The SME is an important parameter to consider during HMMA production as positive correlations between SME and textural attributes (e.g., cutting force, hardness, and chewiness) were previously reported (Chen et al., 2010; Fang et al., 2014; Palanisamy et al., 2019). A similar observation was evident in the current study (Tables 1 and 2) as SME was comparatively higher at lower SFM concentrations, and textural attributes were higher at these conditions. Apart from protein and oil content, the high insoluble dietary fiber content of some raw materials has also been reported as a potential factor for decreased texture attributes of HMMAs, for example, for those produced from blends of SPI and tomato peel powder (Lyu et al., 2023). Since SFM generally contains higher levels of insoluble dietary fiber (~ 53%, d.b.) (Ivanova et al., 2022) than SPI which was mostly composed of protein (~ 88%, d.b.), this may also be responsible for decreased texture attributes, such as hardness, at higher SFM concentrations in the present study.

The results for the degree of texturization are presented in Fig. 1c. The degree of texturization values > 1 generally indicates the presence of fiber-like structures in the direction of flow in the die (Chiang et al., 2019). Overall, the HMMAs produced with blend formulations containing 37.5% and 50% SFM concentration showed a degree of texturization between 1.06 and 1.13 (Fig. 1c). Compared to a previously published study on high-moisture extrusion of SPI and wheat gluten blends at similar barrel temperatures, the degree of texturization values of the SFM-SPI blends in this study are higher than those reported for 100% SPI (0.82), comparable to SPI containing 15% wheat gluten (1.17), and lower than SPI containing 30% wheat gluten (1.60) HMMAs (Wittek et al., 2021). An increase in SFM concentration from 37.5 to 62.5% in the blend formulation generally decreased the degree of texturization (Fig. 1c) which might be attributed to the increased oil content in the 62.5% SFM-containing formula. Higher oil content was reported to be the primary reason responsible for the inability of mechanically pressed SFM to produce a fibrous structure using shear cell technology (Jia et al., 2022a, b). Since the addition of oil decreases the viscosity of the protein melts (Kendler et al., 2021), the flow characteristics of the melt inside the long cooling die are expected to differ as a function of SFM concentration (e.g., the increased velocity at higher SFM concentration due to less resistance to flow). In line with our results, Kendler et al. (2021) reported the formation of dough-like structures at 4% oil content in contrast to fibrous structures that were observed at 0% oil content in wheat gluten-based HMMAs. The degree of texturization of HMMAs produced from blend formulation containing 62.5% SFM at 70% FMC was significantly (*p* < 0.05) lower than all other conditions (Fig. 1c) which may be attributed to their softer and doughy nature. The relatively higher oil content of this blend formulation made the product much softer compared to other conditions. In addition, this blend formulation had the highest variability, based on visual assessment and evidenced by the magnitude of its error bars.

**Fig. 2:**
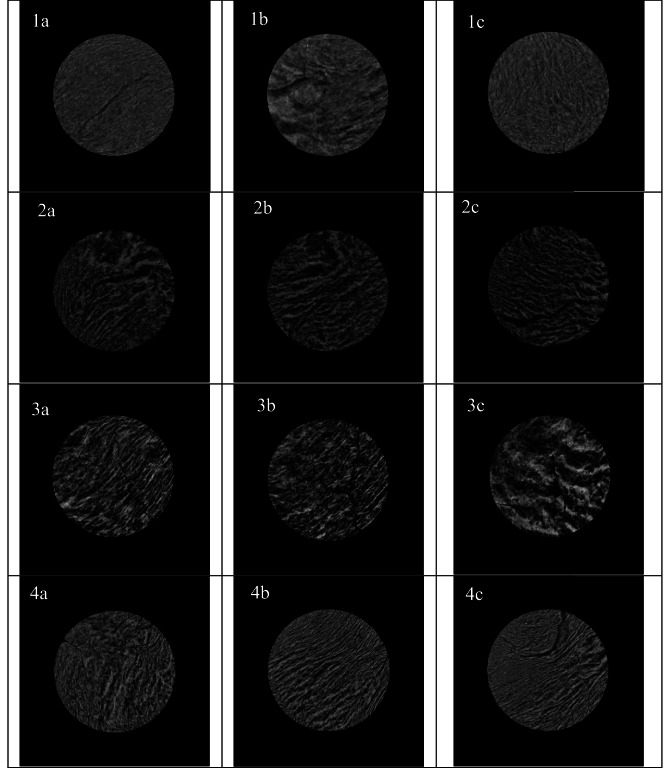

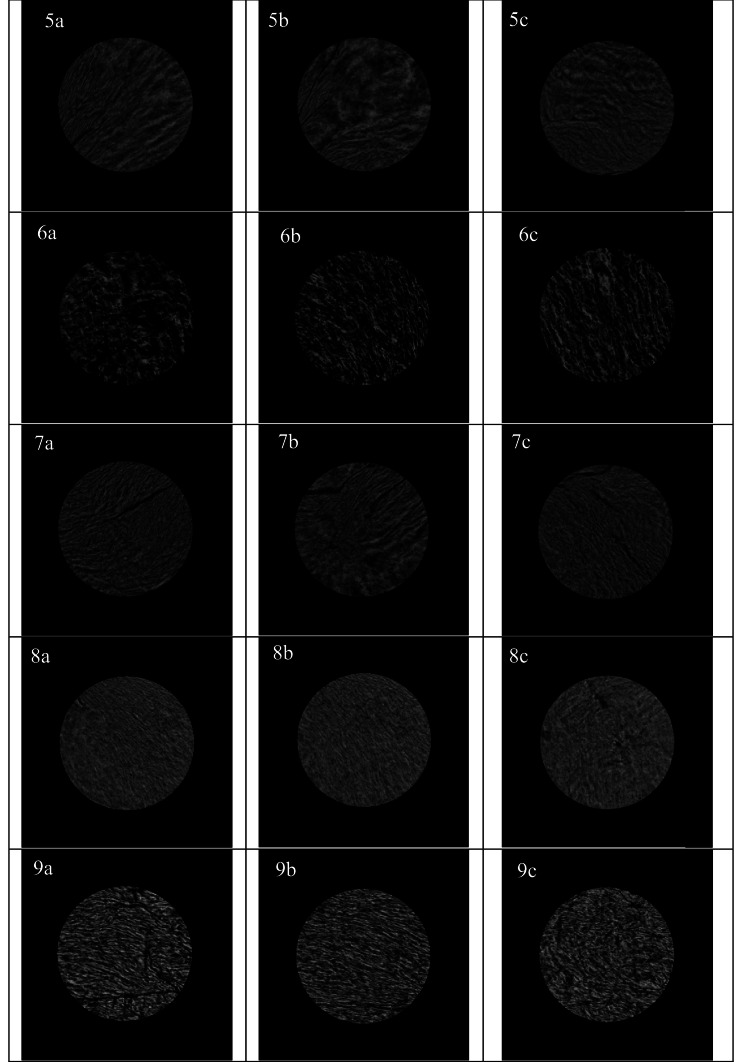
X-ray microcomputed tomography images of freeze-dried HMMA samples: (1) SFM37.5-MC60, (2) SFM37.5-MC65, (3) SFM37.5-MC70, (4) SFM50-MC60, (5) SFM50-MC65, (6) SFM50-MC70, (7) SFM62.5-MC60, (8) SFM62.5-MC65, and (9) SFM62.5-MC70. Three cross-sectional images represent a slice close to the bottom (**a**), center (**b**), and top (**c**) of the freeze-dried HMMA samples. The circles in the images have a diameter of 9 mm

Figure 1 displays the X-ray microtomography images of three cross-sectional slices (top, center, bottom) from freeze-dried HMMAs. All freeze-dried HMMA samples tested showed some level of layered or fibrous structure formation (gray-colored pixels) separated by air (black-colored pixels in the X-ray microtomography images). In line with our findings, the presence of air was reported previously in the shear cell (Jia et al., 2021) and extrusion (Ramos-Diaz et al., 2022) produced HMMAs in fresh and freeze-dried forms, respectively. Furthermore, the observed microstructure of the HMMAs studied suggests that there may be variations as a function of SFM concentration and FMC (e.g., Fig. 2(1a vs. 4a vs. 7a)) and also between the center, top, and bottom of the same treatment (e.g., see Fig. 2(3a vs. 3b vs. 3c)). However, it is important to note that our primary objective in the current study was to perform a preliminary qualitative comparison of the microstructure of the freeze-dried HMMAs rather than a detailed quantitative characterization. These preliminary findings confirm the need for more detailed microstructural analysis, including comparing the porosity of samples under different treatments, to draw more definitive conclusions. Further studies are required to confirm the observed variations in microstructure and to provide stakeholders with more informed decisions about the microstructural quality of the products they produce.

### Color Analysis

The results for color attributes are presented in Table 3. The L* of raw blend formulations significantly decreased for blend formulations containing 62.5% SFM compared to 37.5% SFM, indicating a slightly darker color at higher SFM concentrations. Compared to raw blend formulations, L* significantly (*p* < 0.05) decreased for all the HMMAs studied, i.e., the HMMAs were darker when compared to the raw blends. The decrease in L* is generally due to Maillard reactions or degradation of some bioactive components (e.g., phenolic compounds) during high-temperature processing (Ilo & Berghofer, 1999; Nayak et al., 2011). Interestingly, the HMMAs obtained from 37.5% SFM were generally darker (lower L*) compared to 62.5% SFM; however, this difference was only significant (*p* < 0.05) at 70% FMC. Moreover, the color difference between raw blends with their counterpart HMMAs, i.e., Δ*E*, was significantly (*p* < 0.05) higher for 37.5% SFM compared to 62.5% SFM. The probable reason behind this was the higher mechanical energy (i.e., SME) input that was needed to process the blend formulation containing 37.5% SFM compared to 62.5% SFM (Table 1). Increased mechanical energy has previously been associated with a darker color of soy protein HMMAs (Fang et al., 2014); hence, the results obtained in the present research align with the literature findings. For any SFM concentration studied, an increase in FMC only resulted in significantly (*p* < 0.05) lower Δ*E* at 70% FMC for blend formulation containing 62.5% SFM (Table 3). Palanisamy et al. (2019) also reported a decrease in Δ*E* as a function of FMC for HMMAs produced from a blend of lupin protein concentrate and isolate. Compared to raw blend formulations, the HMMAs were richer in red (higher a*) and deficient in yellow (lower b*) color. Overall, there were no general trends observed for a* and b* as a function of extrusion FMC and SFM concentration.

**Table 3:** Effects of sunflower meal (SFM) concentration and feed moisture content (FMC) on the color attributes, i.e., L*, a*, b*, and Δ*E*, of high-moisture meat analogues

**SFM concentration** **(%)**	**FMC** **(%)**	L*	a*	b*	**ΔE**
**37.5**	**Raw**	79.9 ± 0.1^a^	0.1 ± 0.0^d^	17.9 ± 0.1^a^	**-**
	**60**	39.8 ± 1.2^e^	3.6 ± 0.2^c^	10.5 ± 0.6^bc^	40.9 ± 1.2^ab^
	**65**	39.0 ± 1.0^e^	3.9 ± 0.2^abc^	10.4 ± 0.7^bc^	41.7 ± 1.1^a^
	**70**	39.6 ± 1.0^e^	3.7 ± 0.1^bc^	9.9 ± 0.4^c^	41.2 ± 1.0^a^
**50**	**Raw**	78.8 ± 0.2^ab^	0.2 ± 0.0^d^	17.4 ± 0.1^a^	-
	**60**	40.4 ± 1.2^de^	4.1 ± 0.2^ab^	11.2 ± 0.6^b^	39.1 ± 1.2^bc^
	**65**	41.9 ± 0.6^d^	4.3 ± 0.2^a^	11.5 ± 0.4^b^	37.6 ± 0.7^c^
	**70**	40.5 ± 0.4^de^	3.9 ± 0.2^abc^	10.1 ± 0.6^c^	39.2 ± 0.4^bc^
**62.5**	**Raw**	77.5 ± 0.5^b^	0.3 ± 0.1^d^	17.3 ± 0.3^a^	-
	**60**	40.1 ± 1.5^de^	4.2 ± 0.1^ab^	10.7 ± 0.3^bc^	38.1 ± 1.5^c^
	**65**	41.1 ± 0.5^de^	4.2 ± 0.1^a^	10.5 ± 0.2^bc^	37.2 ± 0.5^c^
	**70**	45.1 ± 1.3^c^	3.9 ± 0.1^bc^	10.9 ± 0.5^bc^	33.2 ± 1.3^d^

Data is expressed as mean ± standard deviation (*n* = 2 for extrusion runs, *n* = 3 for color attributes). Values followed by different letters in a column are significantly different (*p* < 0.05)

One of the major disadvantages of using SFM in food products is the formation of green color due to interactions between sunflower proteins and chlorogenic acid (one of the phenolic compounds present in SFM) (Wildermuth et al., 2016). No such green color was observed for HMMAs produced in the present study, indicating the absence of any protein–chlorogenic acid interactions at the processing conditions used. The absence of such interactions was also previously reported for SFM processed using shear cell technology at 140 °C (Jia et al., 2022a, b). These results indicate the effectiveness of high-moisture extrusion processing for the development of value-added products from SFM without any undesirable effects on their color attributes.

### Amino Acid Composition and Score

The requirement for amino acids is the lowest level of dietary indispensable amino acid intake that will balance the nitrogen losses and maintain protein mass. A different intake level of dietary amino acids is needed to sustain body health for each life stage. Reports from the joint FAO/WHO Expert Consultation on Protein Quality Evaluation (FAO/WHO, 1991, 2013) have established the recommended scoring patterns, such as for preschool children (2 to 5 years) (FAO/WHO, 1991), infants (birth to 6 months), young children (6 months to 3 years), and older children, adolescents, and adults (FAO/WHO, 2013), as presented in Table 4.

**Table 4:** Recommended amino acid (AA) scoring by FAO/WHO for preschool children (2 to 5 years), infants (birth to 6 months), young children (6 months to 3 years), and older children, adolescents, and adults

**AA reference values from FAO/WHO (mg/g protein)**	**His**	**Ile**	**Leu**	**Lys**	**Thr**	**Trp**	**Val**	**Met + Cys**	**Phe + Tyr**
1991, preschool children (2 to 5 years)	19	28	66	58	34	11	35	25	63
2013, for infant (birth to 6 months)	21	55	96	69	44	17	55	33	94
2013, for young children (6 months to 3 years)	20	32	66	57	31	8.5	43	27	52
2013, other children, adolescents, and adults	16	30	61	48	25	6.6	40	23	41

The essential and non-essential amino acid composition of HMMAs is presented in Table 5. The SFM concentration and the FMC did not significantly (*p* < 0.05) affect the composition of essential amino acids (EAA: His, Ile, Leu, Lys, Thr, Trp, Val, Met + Cys, Phe + Tyr), non-essential amino acids (NEAA: Ala, Arg, Asp, Glu, Gly, Pro, Ser), total EAA, and total NEAA. As expected, SFM concentration significantly (*p* < 0.05) impacted the total amino acid content of HMMAs, varying from 58.6 (SFM 62.5%) to 72.3 g/100 g sample (SFM 37.5%). The total EAA content of HMMAs produced in the present study (430–447 mg/g protein) was higher than the reported values for HMMAs produced from SFM (325 mg/g protein), pea protein isolate (386 mg/g protein), and pumpkin seed meal (328 mg/g protein) (Baune et al., 2021).

**Table 5:** Effects of sunflower meal (SFM) concentration and feed moisture content (FMC) on the amino acid composition and amino acid score (AAS) of high-moisture meat analogues

**Amino acid composition**	**High-moisture meat analogues**								
	**SFM 37.5%**			**SFM 50%**			**SFM 62.5%**		
	**FMC 60%**	**FMC 65%**	**FMC 70%**	**FMC 60%**	**FMC 65%**	**FMC 70%**	**FMC 60%**	**FMC 65%**	**FMC 70%**
**Essential AAs (mg/g protein)**									
His	28.8 ± 2.9^a^	28.4 ± 2.7^a^	27.8 ± 0.1^a^	27.3 ± 0.9^a^	27.4 ± 0.8^a^	29.0 ± 1.5^a^	26.4 ± 1.5^a^	27.9 ± 0.7^a^	27.0 ± 2.1^a^
Ile	47.8 ± 0.5^a^	48.5 ± 0.2^a^	49.4 ± 2.0^a^	48.1 ± 0.2^a^	49.8 ± 0.5^a^	46.6 ± 1.4^a^	48.6 ± 0.7^a^	48.3 ± 0.1^a^	49.0 ± 1.4^a^
Leu	74.9 ± 0.6^a^	74.9 ± 0.1^a^	78.2 ± 3.0^a^	74.3 ± 0.2^a^	75.7 ± 1.0^a^	76.1 ± 1.2^a^	74.2 ± 0.2^a^	73.9 ± 0.6^a^	74.4 ± 1.6^a^
Lys	57.5 ± 2.2^a^	57.9 ± 1.9^a^	59.2 ± 2.0^a^	56.7 ± 1.8^a^	57.3 ± 0.1^a^	57.2 ± 0.1^a^	56.9 ± 0.8^a^	56.7 ± 0.1^a^	55.9 ± 1.1^a^
Thr	39.1 ± 1.5^a^	39.1 ± 0.9^a^	39.4 ± 1.0^a^	39.6 ± 0.7^a^	40.5 ± 0.1^a^	38.2 ± 1.1^a^	40.0 ± 0.4^a^	39.9 ± 0.1^a^	40.5 ± 1.2^a^
Trp	12.8 ± 0.9^a^	12.9 ± 0.4^a^	12.8 ± 0.3^a^	12.7 ± 0.3^a^	13.1 ± 0.2^a^	13.3 ± 0.3^a^	13.7 ± 0.1^a^	13.9 ± 0.8^a^	13.5 ± 0.1^a^
Val	48.9 ± 0.4^a^	49.1 ± 0.4^a^	50.1 ± 1.5^a^	49.7 ± 0.2^a^	50.8 ± 0.3^a^	49.3 ± 0.4^a^	50.9 ± 0.2^a^	50.6 ± 0.3^a^	51.3 ± 1.4^a^
Sulfur AAs (Met + Cys)	29.1 ± 2.4^a^	29.4 ± 1.7^a^	29.2 ± 0.9^a^	30.0 ± 0.1^a^	29.1 ± 0.3^a^	31.0 ± 0.5^a^	29.8 ± 0.5^a^	31.8 ± 2.2^a^	31.2 ± 1.0^a^
Aromatic AAs (Phe + Tyr)	97.4 ± 4.2^a^	99.2 ± 5.1^a^	101.0 ± 7.9^a^	97.5 ± 2.1^a^	100.6 ± 1.1^a^	95.3 ± 0.3^a^	94.3 ± 0.8^a^	94.0 ± 1.0^a^	97.9 ± 4.2^a^
**Non-essential AAs (mg/g protein)**									
Ala	42.4 ± 0.6^a^	42.6 ± 0.6^a^	41.8 ± 0.9^a^	43.1 ± 0.6^a^	43.0 ± 0.3^a^	43.1 ± 0.6^a^	43.8 ± 0.1^a^	43.7 ± 0.1^a^	43.7 ± 0.9^a^
Arg	78.8 ± 0.1^a^	79.0 ± 0.3^a^	82.5 ± 4.4^a^	80.2 ± 0.5^a^	82.0 ± 1.3^a^	78.3 ± 2.1^a^	82.2 ± 0.5^a^	81.4 ± 0.2^a^	83.2 ± 1.5^a^
Asp	106.1 ± 1.1^a^	104.0 ± 2.2^a^	98.8 ± 11.1^a^	105.1 ± 0.1^a^	100.6 ± 3.2^a^	108.0 ± 3.5^a^	102.4 ± 0.2^a^	101.9 ± 2.6^a^	99.7 ± 6.0^a^
Glu	189.6 ± 0.1^a^	187.1 ± 1.7^a^	177.9 ± 14.0^a^	186.4 ± 1.9^a^	180.9 ± 1.4^a^	187.4 ± 0.5^a^	187.8 ± 1.9^a^	186.6 ± 1.2^a^	181.8 ± 7.6^a^
Gly	46.4 ± 0.8^a^	47.0 ± 1.4^a^	48.7 ± 3.6^a^	48.6 ± 0.8^a^	49.4 ± 0.8^a^	47.1 ± 0.7^a^	50.5 ± 0.1^a^	50.6 ± 0.2^a^	51.3 ± 1.5^a^
Pro	49.7 ± 1.0^a^	50.0 ± 1.2^a^	51.2 ± 2.3^a^	49.8 ± 0.7^a^	50.2 ± 0.3^a^	48.7 ± 0.2^a^	49.1 ± 0.3^a^	48.8 ± 0.4^a^	49.5 ± 2.0^a^
Ser	50.7 ± 1.0^a^	51.0 ± 0.5^a^	51.9 ± 1.2^a^	50.8 ± 0.1^a^	50.7 ± 0.2^a^	50.5 ± 0.1^a^	49.5 ± 0.5^a^	50.0 ± 1.8^a^	50.2 ± 0.1^a^
**Total essential AAs (mg/g protein)**	436.3 ± 2.4^a^	439.3 ± 0.1^a^	447.1 ± 14.5^a^	436.0 ± 0.2^a^	443.3 ± 2.3^a^	430.0 ± 11.0^a^	434.7 ± 0.8^a^	436.9 ± 0.6^a^	440.6 ± 0.8^a^
**Total non-essential AAs (mg/g protein)**	563.7 ± 2.4^a^	560.7 ± 0.1^a^	552.9 ± 14.5^a^	564.0 ± 0.2^a^	556.7 ± 2.3^a^	567.0 ± 11.0^a^	565.3 ± 0.8^a^	563.1 ± 0.6^a^	559.4 ± 0.8^a^
**Total AAs (g/100 g dry HMMA)**	72.3 ± 4.2^a^	70.6 ± 3.0^a^	68.6 ± 1.4^ab^	66.2 ± 0.9^abcd^	67.3 ± 0.1^abc^	64.0 ± 0.5^abcd^	60.2 ± 0.5^bcd^	59.0 ± 2.8^ cd^	58.6 ± 2.3^d^
**Lowest AAS**^**A**^** (%)**	Lys 99.2 ± 3.7	Lys 99.9 ± 3.3	No deficiency	Lys 97.7 ± 3.1	Lys 98.7 ± 0.1	Lys 98.6 ± 0.1	Lys 98.1 ± 1.4	Lys 97.7 ± 0.2	Lys 96.3 ± 2.0
**Lowest AAS**^**B**^** (%)**	Trp 75.1 ± 5.4	Trp 75.9 ± 2.6	Trp 75.3 ± 1.7	Trp 74.9 ± 1.6	Trp 77.3 ± 1.2	Trp 78.3 ± 1.6	Leu 77.3 ± 0.2	Leu 76.9 ± 0.6	Leu 77.5 ± 1.6
**Lowest AAS**^**C**^** (%)**	No deficiency	No deficiency	No deficiency	No deficiency	No deficiency	No deficiency	Lys 99.8 ± 1.4	Lys 99.4 ± 0.2	Lys 98.0 ± 2.0
**Lowest AAS**^**D**^** (%)**	No deficiency	No deficiency	No deficiency	No deficiency	No deficiency	No deficiency	No deficiency	No deficiency	No deficiency

Data is expressed as mean ± standard deviation (*n* = 2). *AA* amino acid

Values followed by different letters in a row are significantly different (*p* < 0.05)

^A^AAS using FAO/WHO (1991) amino acid scoring pattern for preschool children (2 to 5 years)

^B^AAS using FAO/WHO (2013) amino acid scoring pattern for infants (birth to 6 months)

^C^AAS using FAO/WHO (2013) amino acid scoring pattern for young children (6 months to 3 years)

^D^AAS using FAO/WHO (2013) amino acid scoring pattern for older children, adolescents and adults

Furthermore, according to FAO/WHO (1991) preschool children requirements as the reference, lysine was the first-limiting amino acid for all samples except for the HMMA produced from the formulation containing 37.5% SFM at 70% FMC which showed no deficiency. Baune et al. (2021) also reported lysine as the first-limiting amino acid for HMMAs produced from SFM; however, the AAS of their HMMA (AAS: 69) was lower than the AAS of all HMMAs produced in the current study (Table 3). This reflects the effectiveness of soy protein and SFM blending to produce HMMAs with relatively better amino acid profiles. Based on the FAO/WHO (2013) scoring pattern for infants, tryptophan and leucine were the first-limiting amino acids, while for young children, only the SFM 62.5% HMMAs presented lysine deficiency. No deficiency was shown in samples when the FAO/WHO (2013) for older children, adolescents, and adults scoring pattern was used. These results demonstrate the importance of appropriate reference patterns since reference values inherently modify the overall amino acid score, the first-limiting amino acid, and IV–PDCAAS, also implicating the establishment of protein content claims within jurisdictions.

### Protein Content, In Vitro Protein Digestibility, IV–PDCAAS, and IV–DIAAS

Table 6 presents the protein content (%, d.b.), in vitro protein digestibility (IVPD), in vitro–protein digestibility corrected amino acid score (IV–PDCAAS), and in vitro–digestible indispensable amino acid score (IV–DIAAS) of the HMMAs. Protein content (d.b.) varied from 63.2% (in HMMAs containing 62.5% SFM) to 74.5% (in HMMAs containing 37.5% SFM), while FMC did not significantly (*p* < 0.05) affect the protein content of HMMAs on a dry basis. Similar to the protein content of HMMAs in this study, Zahari et al. (2021) produced HMMAs from a blend of rapeseed protein concentrate and pea protein isolate with a protein content of 72.8% (d.b.), and Ramos-Diaz et al. (2022) produced HMMAs from blends of oat fiber concentrate and pea protein isolate having protein content in the range of 40.4 and 71.5% (d.b.).

**Table 6:** Effects of sunflower meal (SFM) concentration and feed moisture content (FMC) on the protein content, in vitro protein digestibility (IVPD), in vitro–protein digestibility corrected amino acid score (IV–PDCAAS), and in vitro–digestible indispensable amino acid score (IV–DIAAS) of high-moisture meat analogs

**SFM concentration** **(%)**	**FMC** **(%)**	**Protein content** **(%, d.b.)**	**IVPD** **(%)**	**IV–PDCAAS**^**A**^** (%)**	**IV–DIAAS**^**B**^** (%)**	**IV–DIAAS**^**C**^** (%)**	**IV–DIAAS**^**D**^** (%)**
**37.5**	**60**	74.5 ± 0.1^a^	90.8 ± 0.4^a^	90.1 ± 3.9^a^	68.2 ± 5.2^a^	90.8 ± 0.5^a^	90.8 ± 0.5^a^
	**65**	74.0 ± 0.2^a^	90.3 ± 0.4^a^	90.2 ± 2.7^a^	68.6 ± 2.2^a^	90.3 ± 0.3^a^	90.3 ± 0.3^ab^
	**70**	74.0 ± 0.1^a^	90.8 ± 0.5^a^	90.8 ± 0.1^a^	68.4 ± 1.6^a^	90.8 ± 0.1^a^	90.8 ± 0.1^a^
**50**	**60**	68.5 ± 0.2^b^	89.7 ± 0.6^ab^	87.6 ± 3.0^a^	67.2 ± 1.3^a^	89.7 ± 0.3^a^	89.7 ± 0.3^abc^
	**65**	68.6 ± 0.3^b^	89.5 ± 0.8^abc^	88.4 ± 0.9^a^	69.2 ± 1.7^a^	89.5 ± 0.8^a^	89.5 ± 0.8^abc^
	**70**	69.1 ± 0.2^b^	88.8 ± 0.8^ cd^	87.6 ± 0.3^a^	69.5 ± 1.6^a^	88.8 ± 0.3^ab^	88.8 ± 0.3^bc^
**62.5**	**60**	63.3 ± 0.2^c^	88.8 ± 0.6^ cd^	87.1 ± 0.9^a^	68.6 ± 0.5^a^	88.6 ± 0.9^ab^	88.8 ± 0.4^bc^
	**65**	63.2 ± 0.2^c^	88.8 ± 0.6^ cd^	86.8 ± 0.4^a^	68.3 ± 0.1^a^	88.3 ± 0.4^ab^	88.8 ± 0.6^bc^
	**70**	63.8 ± 0.5^c^	88.1 ± 0.5^d^	84.8 ± 1.6^a^	68.3 ± 1.4^a^	86.3 ± 1.6^b^	88.1 ± 0.1^c^

Data is expressed as mean ± standard deviation (*n* = 3)

Values followed by different letters in a column are significantly different (*p* < 0.05)

^A^IV–PDCAAS was calculated: IVPD × AAS [using FAO/WHO (1991) amino acid scoring pattern for preschool children (2 to 5 years)]

^B^IV–DIAAS in this row was calculated: IVPD × AAS [using FAO/WHO (2013) amino acid scoring pattern for infants (birth to 6 months)]

^C^IV–DIAAS in this row was calculated: IVPD × AAS [using FAO/WHO (2013) amino acid scoring pattern for young children (6 months to 3 years)]

^D^IV–DIAAS in this row was calculated: IVPD × AAS [using FAO/WHO (2013) amino acid scoring pattern for older children, adolescents and adults]

IVPD of the HMMAs varied from 88.1% (in HMMAs containing 62.5% SFM) to 90.8% (in HMMAs containing 37.5% SFM), while FMC did not significantly (*p* < 0.05) affect the HMMA IVPD. Xie et al. (2022) studied the in vitro digestibility of meat (e.g., pork and beef) and plant-based meat analogues (e.g., soybean, pea, and rice protein-based pork alternative and pea, rice, and mung bean protein-based beef alternative) using a static protocol (Brodkorb et al., 2019). The results of in vitro digestion for pork and beef were ~ 80% digestibility, while plant-based beef and plant-based pork alternatives showed ~ 60% and ~ 50% digestibility, respectively. The differences in the in vitro digestion were explained by (i) the plant-based alternatives’ protein having a different secondary structure (i.e., lower* α*-helices and *β*-sheets but higher *β*-turns and random coils than the animal protein), (ii) their higher viscosity that may reduce the reaction rate by affecting the contact between the protein and the digestive enzymes, and (iii) their starch content (Xie et al., 2022).

The IV–PDCAAS of HMMAs varied from 84.8 to 90.8% and was not significantly (*p* < 0.05) affected by the SFM concentration and FMC. The IV-PDCAAS of HMMAs produced is higher than the reported values for HMMAs produced from SFM (65%), pea protein isolate (56%), and pumpkin seed meal (49%) (Baune et al., 2021). Literature showed the IV–PDCAAS results for oilseed meals, such as raw cold-pressed sesame seed meal (70.9%) (Sá et al., 2022), which is lower than those presented in this study.

HMMAs, with the lowest AAS and IVPD using scoring patterns for infants (birth to 6 months), young children (6 months to 3 years), and older children, adolescents, and adults (FAO/WHO, 2013), had different IV–DIAAS scores (Table 6). For infants, IV–DIAAS ranged between 67.2–69.5% with no significant (*p* < 0.05) effect of SFM concentration and FMC. The IV–DIAAS for young children varied from 86.3% (in HMMAs containing 62.5% SFM) to 90.8% (in HMMAs containing 37.5% SFM), and for older children, adolescents, and adults, the results ranged between 88.1–90.8% where higher SFM concentrations led to slightly lower IV–DIAAS values. Furthermore, when comparing an HMMA sample for the same age category, i.e., comparing the IV–PDCAAS using 2–5 years (FAO/WHO, 1991) reference pattern and IV–DIAAS using younger children (6 months to 3 years) (FAO/WHO, 2013), the results were similar (Table 6). As the same protein digestibility and amino acid profile were used for IV–PDCAAS and different IV–DIAAS calculations, the similarity between these values occurs specifically due to the amino acid reference pattern, particularly for lysine, which was the first-limiting amino acid of HMMAs studied (IV–PDCAAS requirement for Lys is 58 mg/g protein, similar to the IV–DIAAS requirement for Lys for young children (similar age category): 57 mg/g protein—as presented in Table 4).

Although animal experimentation (e.g., in vivo PDCAAS) is still required by governmental regulations for the determination of protein quality, recent studies suggest strong correlations (*R*^2^: 0.7497–0.9971) between in vivo and in vitro measurements of protein digestibility and protein quality for different plant-based sources (Nosworthy & House, 2017; Nosworthy et al., 2017, 2018a, b; Tavano et al., 2016). Therefore, these strong correlations suggest that in vitro PDCAAS for determining protein quality could be used as a surrogate for in vivo evaluation of plant-based protein ingredients. In addition, in vitro approaches showed advantages over in vivo assays, such as convenience, simplicity, lower cost, speed, and especially reduced animal usage (Tavano et al., 2016). There are limitations to using in vitro protein digestibility as a proxy for fecal and ileal amino acid digestibility; however, other limitations exist, especially regarding the sample amount available for DIAAS and PDCAAS analysis. Although alternative approaches are being developed for in vitro amino acid digestibility assessments, these methods are still in their infancy, particularly for plant-based protein sources.

## Conclusion

SFM was successfully incorporated in extrusion blend formulations containing SPI at three different levels i.e., 37.5%, 50%, and 62.5% (w/w) to produce HMMAs. The formation of fibrous textures in the direction of flow was reflected in the degree of texturization values that were greater than 1. HMMAs with softer textures were produced when higher SFM concentration or FMC was used. An increase in SFM concentration and FMC also reduced the mechanical energy inputs required for extrusion processing. Although quantitative analyses are needed for further insights, preliminary qualitative X-ray microstructural analyses showed variations in microstructural attributes of HMMAs as a function of SFM concentration and FMC.

The blending of SFM and SPI proved to be highly efficient for producing HMMAs with relatively well-balanced amino acid profiles as AAS of HMMAs containing up to 50% SFM showed no amino acid deficiency when the FAO/WHO (2013) scoring patterns for young children, older children, adolescents, and adults were used. Similarly, HMMAs also had high IVPD (88–90%) and IV-PDCAAS values (85–91%). In general, the addition of SFM up to 50% showed no negative effects on any of the protein quality attributes studied. The importance of using appropriate amino acid scoring patterns was also evidenced as the lowest AAS showed variations for different age categories. Overall, these results indicate that SFM has a great potential for partly (up to 50%) replacing SPI for the production of fibrous HMMAs without negatively affecting their physical and nutritional quality. This research work will help in providing new opportunities for re-introducing SFM into novel meat alternatives, thereby adding value to this nutritious oil industry by-product. Future work will investigate the effects of extrusion processing conditions on the in vivo protein quality of SFM-based HMMAs that will help gather pivotal information for protein content claims. The microstructural quality of SFM-based HMMAs will also be investigated quantitatively to explore the microstructural characteristics of HMMAs and link them to textural quality attributes.

## Data Availability

Data associated with the research shall be made available on reasonable request.
